# Editorial: The use of data mining in radiological-pathological images for personal medicine

**DOI:** 10.3389/fgene.2023.1187040

**Published:** 2023-03-27

**Authors:** Jincheng Wang, Xudong Zhang, Jinhui Liu, Yin Yin

**Affiliations:** ^1^ Department of General Surgery, The First Affiliated Hospital of Anhui Medical University, Hefei, China; ^2^ Department of Hepatobiliary Surgery, The Affiliated Drum Tower Hospital of Nanjing University Medical School, Nanjing, China; ^3^ The First Affiliated Hospital of Nanjing Medical University, Nanjing, China; ^4^ Department of Hepato-Biliary-Pancreatic Surgery, The Affiliated Changzhou, No.2 People’s Hospital of Nanjing Medical University, Changzhou, China

**Keywords:** data mining, medical image, deep learning, image finding, personalized medicine

The use of data mining in radiological-pathological images for personalized medicine is a promising and rapidly developing field. This innovative approach involves the integration of large amounts of data from medical images, pathology reports, and clinical records to improve patient care and treatment outcomes.

Currently, there are mainly two methods for data mining on medical images: image findings (subjectively summarized factors) (Renzulli et al., 2016; Yue et al., 2022) and high-order features (objectively calculated features, such as radiomics and pathomics) (Lambin et al., 2017; Chen et al., 2022). Image findings can be easily obtained and explained by pathophysiological mechanisms (Segal et al., 2007). However, they are determined by readers and stability need to be improved. Fortunately, deep learning techniques can behave like an experienced reader and compute more robust evaluation results (Zhao et al., 2020). High-order features depend on image analysis techniques which attracted great attention in the last few years. Most research focused on tumor characteristics and prognosis (Xu et al., 2019; Ji et al., 2020; Hu et al., 2022; Xu et al., 2022), and chronic disease can also be fully detected (Wang et al., 2020; Zhang et al., 2022a; Zhang et al., 2022b; Wang et al., 2022). Different from image findings, it is hard to explain biological role of each high-order feature which is worthy of more exploration.

We believe articles published in this Research Topic can provide more evidence for data mining on medical image in personalized medicine. Despite the many benefits of data mining in radiological-pathological images, there are also some potential challenges and risks to be aware of. One of the biggest challenges is ensuring that the data being used is accurate, reliable, and representative of the patient population being studied. Additionally, there are concerns about privacy and data security, particularly as medical records and images contain sensitive information about patients.

Overall, the use of data mining in radiological-pathological images for personalized medicine is an exciting development that has the potential to transform the way we diagnose and treat diseases. With careful attention to data quality and patient privacy, this approach has the potential to help clinical decision-making and improve patient outcomes.
